# Genome-wide identification and analysis of *Oleosin* gene family in four cotton species and its involvement in oil accumulation and germination

**DOI:** 10.1186/s12870-021-03358-y

**Published:** 2021-12-04

**Authors:** Yanchao Yuan, Xinzhe Cao, Haijun Zhang, Chunying Liu, Yuxi Zhang, Xian-Liang Song, Shupeng Gai

**Affiliations:** 1 College of Life Sciences, Qingdao Agricultural University, Key Lab of Plant Biotechnology in Universities of Shandong Province, Qingdao, China; 2 State Key Laboratory of Crop Biology/Agronomy College, Shandong Agricultural University, Taian, Shandong China

**Keywords:** Cotton, Oleosin, Gene family, Gene identification, Fatty acid, Seed germination

## Abstract

**Background:**

Cotton is not only a major textile fiber crop but also a vital oilseed, industrial, and forage crop. Oleosins are the structural proteins of oil bodies, influencing their size and the oil content in seeds. In addition, the degradation of oleosins is involved in the mobilization of lipid and oil bodies during seed germination. However, comprehensive identification and the systematic analysis of the *Oleosin* gene (*OLEOs*) family have not been conducted in cotton.

**Results:**

An in-depth analysis has enabled us to identify 25 and 24 *OLEOs* in tetraploid cotton species *G. hirsutum* and *G. barbadense,* respectively, while 12 and 13 *OLEOs* were identified in diploid species *G. arboreu*m and *G. raimondii,* respectively. The 74 *OLEOs* were further clustered into three lineages according to the phylogenetic tree. Synteny analysis revealed that most of the *OLEO*s were conserved and that WGD or segmental duplications might drive their expansion. The transmembrane helices in GhOLEO proteins were predicted, and three transmembrane models were summarized, in which two were newly proposed. A total of 24 candidate miRNAs targeting *GhOLEOs* were predicted. Three highly expressed oil-related *OLEOs*, *GH_A07G0501* (SL), *GH_D10G0941* (SH*)*, and *GH_D01G1686* (U*)*, were cloned, and their subcellular localization and function were analyzed. Their overexpression in Arabidopsis increased seed oil content and decreased seed germination rates.

**Conclusion:**

We identified OLEO gene family in four cotton species and performed comparative analyses of their relationships, conserved structure, synteny, and gene duplication. The subcellular localization and function of three highly expressed oil-related OLEOs were detected. These results lay the foundation for further functional characterization of *OLEOs* and improving seed oil content.

**Supplementary Information:**

The online version contains supplementary material available at 10.1186/s12870-021-03358-y.

## Background

Cotton (*Gossypium. spp.*) seed is the sixth-largest vegetable oil resource globally and the renewable raw materials for biofuel and industrial products, such as hydraulic oils and lubricants [1]. The demand for biodiesel is growing, whereas cottonseed oil accounts for only 17–27% of seed weight [2]. Therefore, increasing the oil content becomes an important target for cotton breeding. Nowadays, genes related to lipid biosynthesis have been studied extensively. Thus, the homology genes involved in the lipid storage or metabolism pathway should be analyzed to understand functional differentiation.

The seed lipids are mainly stored as triacylglycerols (TAGs) in oil bodies (OBs), which are lipid storage organelles and are widely distributed in lipid storage cells [3]. The oil body is approximately 0.5-2 μm in diameter and has the basic structure that a core of neutral lipids surrounded by the phospholipid monolayer with specific proteins [4]. It is well known that the OBs are synthesized in the endoplasmic reticulum (ER) and then budded to the cytoplasm [4, 5]. The specific proteins in oil bodies, mainly oleosin, caleosin, and steroleosin, have been shown to play an essential role in regulating oil body size and number and lipid accumulation [5, 6]. Among them, oleosins account for 80-90% of the structural proteins in OBs and play a key role in lipid storage [7]. The *Oleosin* (*OLEO*) gene sequence was first identified and characterized in maize [8] and then cloned in various plants, including rapeseed [9], sesame [10], soybean [11], rice [12], sunflower [13].

*OLEOs* from diverse plants can be divided into six lineages (M, T, P, U, SL, and SH) [14]. The M (Mesocarp) lineage exists in the *Lauraceae*, and the T (Tapetum) is only identified in the Tapeta of *Brassicaceae*. The P (Primitive) lineage was mainly distributed in green algae, which might be the origin of the U (Universal) lineage detected in all terrestrial plants, and further evolved to SL (Seed low-molecular-weight) and SH (Seed high-molecular-weight) lineages [14–16]. In *Arabidopsis thaliana*, 17 *OLEOs* were identified, including nine of T, three of U, two of SL, and three of SH [17].

Oleosin peptides can be divided into three domains: a central hydrophobic domain (72 residues), an N-terminal amphiphilic region (50-70 residues), and a C-terminal amphiphilic region (variable length). The central hydrophobic domain is a highly conserved hairpin structure that can insert into the phospholipid membrane and plays an essential role in targeting and stabilizing the oil body [18]. The loop of the hairpin is a proline junction (PX_5_SPX_3_P, X representing a large nonpolar residue), in which the Pro and Ser residues are highly conserved in various plants. The replacement of Pro residue can lead to abnormal targeting of the OB [19]. The N- and C-terminal amphiphilic domains, located on the surface of the oil body, can not only maintain the size of the OB through their steric hindrance and electronegative repulsion [20] but also regulate the OB by combining it with metabolic enzymes and regulatory proteins [21–23]. Take together, oleosins are involved in the oil bodies regulation and biosynthesis, and metabolism of lipid [24, 25].

As lipid storage organelles, the number and size of oil bodies are partly related to the oil content of seeds, and high-oil seeds tend to have more oil bodies [26]. Furthermore, many previous studies showed that oleosins affect the size and number of seed oil bodies and regulate the oil content [17, 27–31]. In the middle stage of seed development, the lack of oleosins caused oil body fusion, leading to larger oil bodies and the decrease of oil content [7, 32]. Similar results showed that the overexpression of *OLEO1* and *OLEO4* was found to reduce the size of oil bodies in *A. thaliana* seeds [33]. Additionally, the overexpression of *GmOLEO1* increased the number of oil bodies and oil accumulation, whereas the OB size decreased [31]. Moreover, co-expression of *OLEOs* and the genes involved in TAG biosynthesis increased lipid production [34], which might be a new method to enhance oil content more efficiently.

It is also reported that the *OLEOs* could be induced by water stress, jasmonic acid, ABA, and osmotic stabilizer [35], indicating that *OLEOs* might play an essential role in engineering salt, cold, and drought tolerance in plants. Shimada et al. (2008) found that the *OLEOs* could maintain the germination rate (GR) of Arabidopsis seeds and improve their overwintering viability by inhibiting the oil body fusion. In addition, *OLEOs* were also involved in heat-shock in embryogenic carrot cell lines [36] and induced by ABA, salt, and PEG in sorghum, suggesting that oleosin proteins could regulate the stability and permeability of the membrane [37].

Oleosins have been widely studied nowadays, whereas little attention has been paid to the *OLEO* gene family in critical oil crops. In the current study, the oleosins from the four cotton species, *G. arboreum*, *G. raimondii*, *G. barbadense*, and *G. hirsutum*, were identified. Their features, including gene structure, conserved motifs and domain, chromosomal location, evolution, subcellular, synteny relationship, and expression patterns, were analyzed and characterized. In addition, the transmembrane helices in GhOLEO proteins were predicted and summarized into three transmembrane models. Subsequently, some *GhOLEOs* preferentially expressed in ovules were cloned and transformed into Arabidopsis. The overexpression of *OLEOs* promoted the accumulation of lipid in Arabidopsis seeds and decreased seed germination rates. Overall, these results provide a foundation for further understanding the function of oleosins in cotton.

## Results

### Whole-genome identification and characterization of *Oleosin* genes in four cotton species

Combined with the results of BLASTP search, HMMER analysis, and CDD check, a total of 25, 24, 12, and 13 *Oleosins* were identified in two tetraploids, *G. hirsutum* and *G. barbadense*, and two diploids, *G. arboreum* and *G. raimondii*, respectively. The gene ID, physical location, and other features of these *Oleosins* were listed in Table S1. The number of amino acids (NAA), molecular weight (Mw), and isoelectric point (pI) of oleosin proteins varied from 116 to 237, 12.30 kDa to 26.47 kDa, and 8.48 to 10.97 in *G. arboreum*, respectively. Those were 116 to 237, 12.42 kDa to 26.55 kDa, and 8.72 to 11.30 in *G. raimondii*, respectively, and similar to *G. arboreum*. In addition, the NAA, Mw, and pI of oleosin proteins in A-genomes of *G. hirsutum* were 116-175, 12.33-18.49 kDa, and 9.36-10.97, respectively, which were similar to those of oleosin proteins in A-genomes of *G. barbadense*, with the value ranges were 116-175, 12.33-18.54 kDa, and 9.36-10.97, respectively. The NAA, Mw, and pI of oleosin proteins in the D-genomes of *G. hirsutum* were also similar to those in the D-genomes of *G. barbadense*. In contrast, those characters of diploids (*G. arboreum* and *G. raimondii*) were not similar to tetraploids (*G. hirsutum* and *G. barbadense*), which mainly were caused by *Ga02G0988* and *Gorai.002G174000*. These results suggested that two diploids might be subjected to one same or similar environmental pressure. In contrast, two tetraploids might be subjected to another similar selection pressure.

The number of *OLEOs* in the A- and D-genomes is nearly equal in all four cotton species. The *OLEO* numbers in the A-genome of *G. arboreum*, *G. hirsutum*, and *G. barbadense* were all 12, while that in the D-genome of *G. raimondii*, *G. hirsutum*, and *G. barbadense* were 13, 13, and 12, respectively. As shown in Fig. S1, there were two *OLEOs* on each Chr05/A05/D05 and Chr09/A09/D09, and one *OLEO* on each Chr06/A06/D06, Chr10/A10/D10, Chr11/A11/D11, Chr12/A12/D12, and Chr13/A13/D13, in *G. arboreum*/*G. hirsutum*/*G. barbadense* respectively. It was different that one and three Oleosins were in Chr02 and Chr07 of *G. arboreum*, respectively, while one and two Oleosins in A01/D01 and A07/D07 of *G. hirsutum* and *G. barbadense*, respectively. These results indicated that Oleosins had a similar distribution on chromosomes in *G. arboreum*, *G. hirsutum,* and *G. barbadense*. Moreover, the *OLEOs* in *G. raimondii* were located in chromosome Chr01, Chr02, Chr06, Chr07, Chr08, Chr09, Chr10, Chr11, and Chr13 with numbers were three, one, two, one, one, two, one, one, and one, respectively. The *OLEO* distributions on chromosomes in *G. raimondii* were different from the other three species. These results were in agreement with those of predecessors that A-genome of *G. arboreum* and *G. hirsutum* originate from a common ancestor A0 [38], and the nascent allotetraploid, which originated from hybridization of an A- and D-genome-like species, diverged into five cotton species, including *G. barbadense* and *G. hirsutum* [39–41]*.*

### Phylogenetic analysis of *Oleosin* genes in four cotton species

**Fig. 1:**
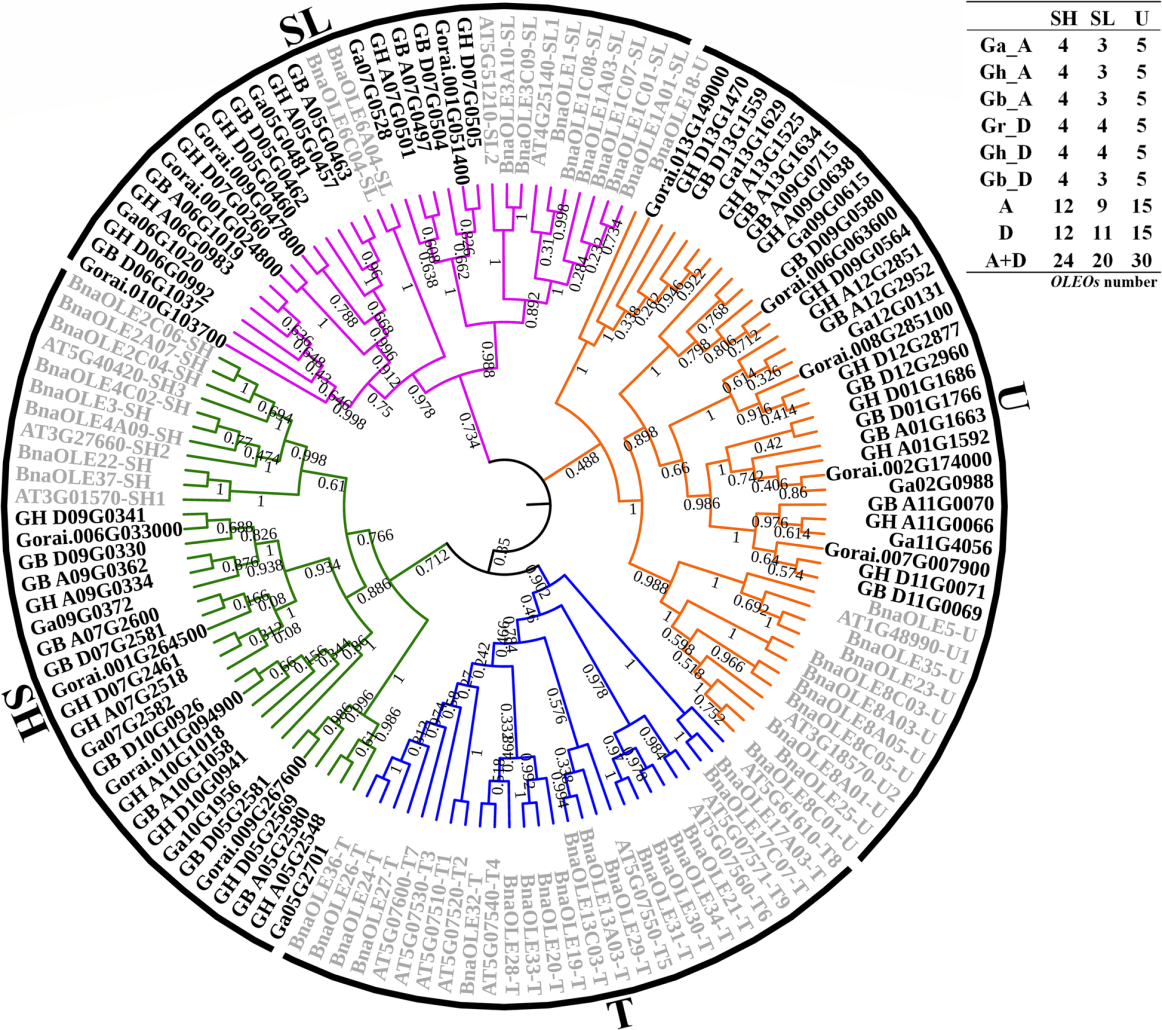
Phylogenetic tree of *Oleosin* genes from four cotton species, *Arabidopsis* and *Brassica napus*. The bootstrap values from 0.08 to 1 were shown on the branch lines

To study the phylogeny and subgroups of the *OLEO* family, a phylogenetic tree was constructed by all oleosin protein sequences in four cotton species combined with 17 *A. thaliana* and 48 *B. napus* oleosin proteins (Fig. 1). In the phylogenetic tree, the cotton *OLEOs* were divided into three lineages (SH, SL, and U) without T lineages, which were only founded in the Tapeta of *Brassicaceae* [14]. In detail, the four species had the same number of each subclass of oleosins per haploid genome with the exception of SL class, for which there were three copies in *G barbadense* and *G. arboretum*, but four copies in *G. raimondii* and the D genome of *G. hirsutum* (Fig. 1). Furthermore, two genes from each tetraploid species and one gene from each diploid species cluster together in each branch of the phylogenetic tree, except *Gorai.001G024800* and *GH_D07G0260* in SL. The two genes from each tetraploid species in each branch were one A-genome and one D-genome gene. This might be due to the fact that tetraploid is derived from two diploids [42] or that the subgenomes of tetraploids and diploids are derived from the same ancestors [38].

### Synteny and duplication analysis of *Oleosin* genes

To show the synteny relationships, the whole genomes and all the homologous *OLEOs* of the four cotton species were aligned and analyzed (Table S2; Fig. S2). Of all the 25 *GhOLEOs*, 23, 24, and 25 had homologous *OLEOs* in *G. arboreum*, *G. raimondii,* and *G. barbadense*, respectively, and 23 *GhOLEOs* were the common homologous genes in the other three cotton species.

During *Gossypium* evolution, the duplicate mechanisms, including tandem, proximal and dispersed duplications, and WGD (whole-genome duplications) or segmental duplications, play an important role in the expansion of gene families [43, 44]. In the tetraploid kinds of cotton (*G. hirsutum* and *G. barbadense*), the duplications of *OLEO*s were all categorized as WGD or segmental duplications (Table S3). However, in the diploids, three *GaOLEOs* (*Ga05G2701*, *Ga07G0528*, and *Ga13G1629*) and four *GrOLEOs* (*Gorai.001G051400*, *Gorai.009G267600*, *Gorai.011G094900*, and *Gorai.013G149000*) were considered as dispersed duplications, while the others were all the WGD or segmental duplications. Therefore, the expansion and evolution of the *OLEO* gene family in cotton might be mainly driven by WGD or segmental duplications.

**Fig. 2:**
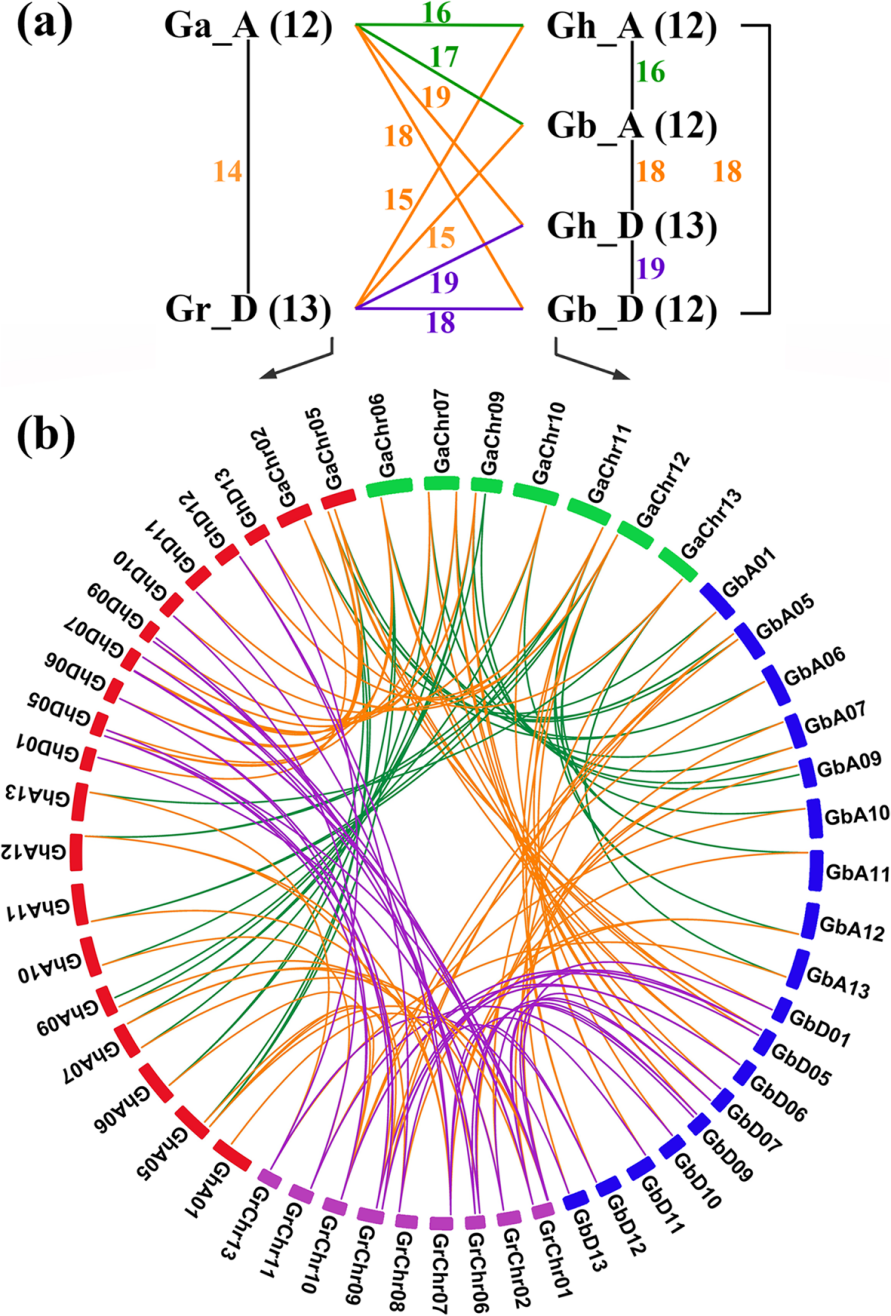
Synteny of *Oleosin* genes among *G. hirsutum*, *G. barbadense*, *G. arboretum* and *G. raimondii*. **a** The number of collinear gene pairs among the four cotton cultivars. **b** Visualization of *Oleosin* genes synteny

For understanding the collinear relationships of all the cotton *OLEO* genes among the two tetraploids and two diploids, the linked gene pairs were all identified (Fig. S2 and Fig. 2). Corresponding to *G. arboreum*, there were 16 and 17 collinear gene pairs identified in the A-genomes of *G. hirsutum* and *G. barbadense*, respectively, and 19 and 18 pairs were identified in the D-genomes of *G. hirsutum* and *G. barbadense*, respectively. In addition, corresponding to *G. raimondii*, 15 and 15 collinear gene pairs were identified in the A-genomes of *G. hirsutum* and *G. barbadense*, respectively, and 19 and 18 pairs were in the D-genomes of *G. hirsutum* and *G. barbadense*, respectively. Furthermore, the gene pairs number between tetraploid A-genomes and diploid genomes were approximate, and the same between tetraploid D-genomes and diploid genomes.

### Gene structure and conserved motif analysis of *Oleosin* genes

**Fig. 3:**
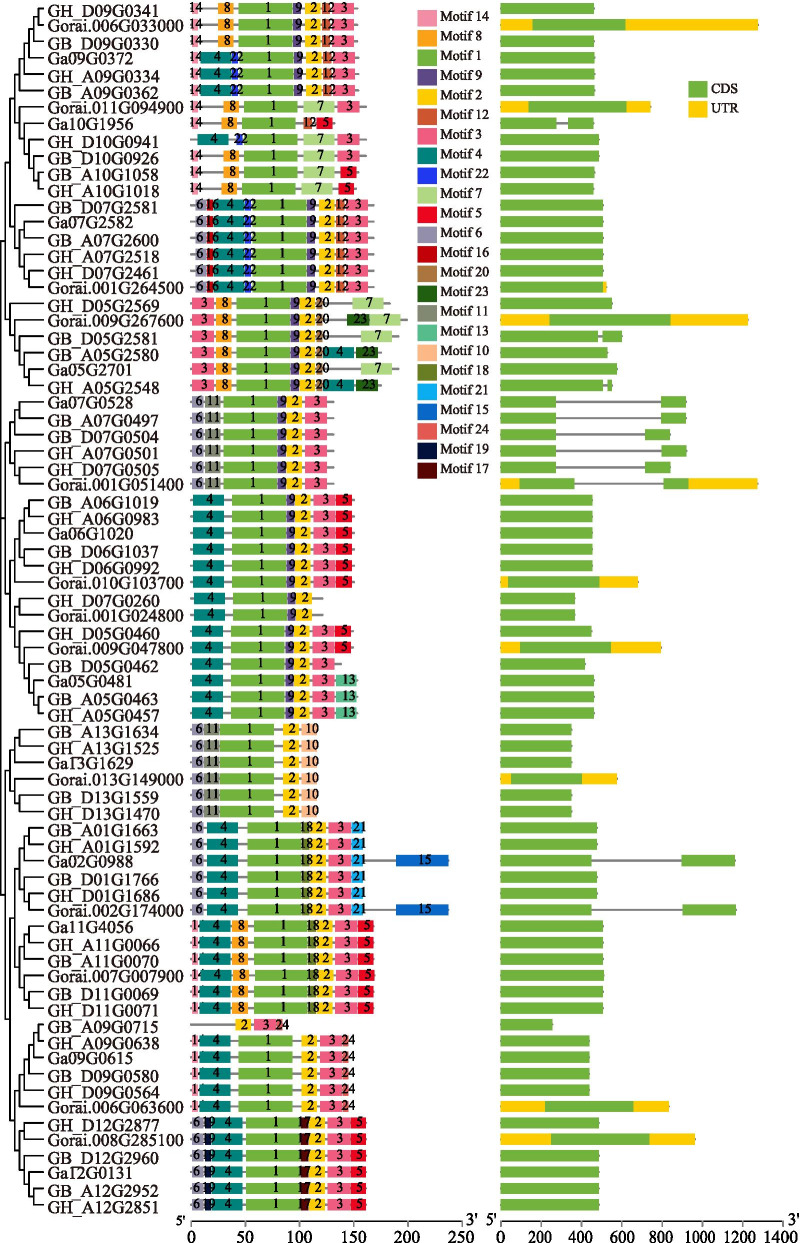
Conserved motif and gene structure of *Oleosin* genes in four cotton species

Based on the gene annotation gff3 files, the gene structure of *OLEO* genes was analyzed and showed in Fig. 3. Results showed that the great majority of the *OLEO* genes had only one exon, and 11 genes contained two exons and one intron. Furthermore, the genes in the branch of the same subfamily contained similar gene structures such as intron/exon number and intron/ exon length. In addition, ten of thirteen *OLEO* genes in *G. raimondii* had the untranslated regions (UTRs), and there were no UTRs in *OLEOs* of the applied genome versions of *G. arboreum*, *G. hirsutum* and *G. barbadense* in this study, because their gff3 files had no UTR annotations.

The conserved motifs were analyzed with the full-length oleosin protein sequences submitted to MEME web-server. As shown in Fig. 3 and Table S4, 24 conserved motifs with 6 - 50 amino acids were identified in *OLEO* genes. The number of conserved motifs in *OLEO* genes ranged from three to nine, and only one gene had three motifs, and two genes had four motifs. Motif 1- 6 were all founded in the SH, SL, and U *OLEOs*. Motif 1, which containing proline junction (PX_5_SPX_3_P), was common to almost all *OLEO* genes, and only one gene, *GB_A09G0715*, did not contain motif 1 but had a proline junction. Following motif 1, motif 2 and motif 3 were conserved in 68 and 63 *OLEOs*, respectively. Thus, the number and type of conserved motifs were similar in the same branch of the evolutionary tree, and some motifs were specific to subfamilies. For example, motifs 10, 15, 17, 18, 19, 21, and 24 were specific to subfamily U, while motifs 7, 12, 13, 16, 20, 22, and 23 were exclusively in SH subfamily members. These indicated that the proteins in different subfamilies had different conserved motif distributions, which might provide a reference for studying the functional differentiation between subfamilies. In the same subfamily, the conserved motifs distribution and gene structure were similar. They proved the accuracy of the evolutionary tree constructed in this study from another aspect.

### The miRNA targeting *GhOLEO* genes

**Fig. 4:**
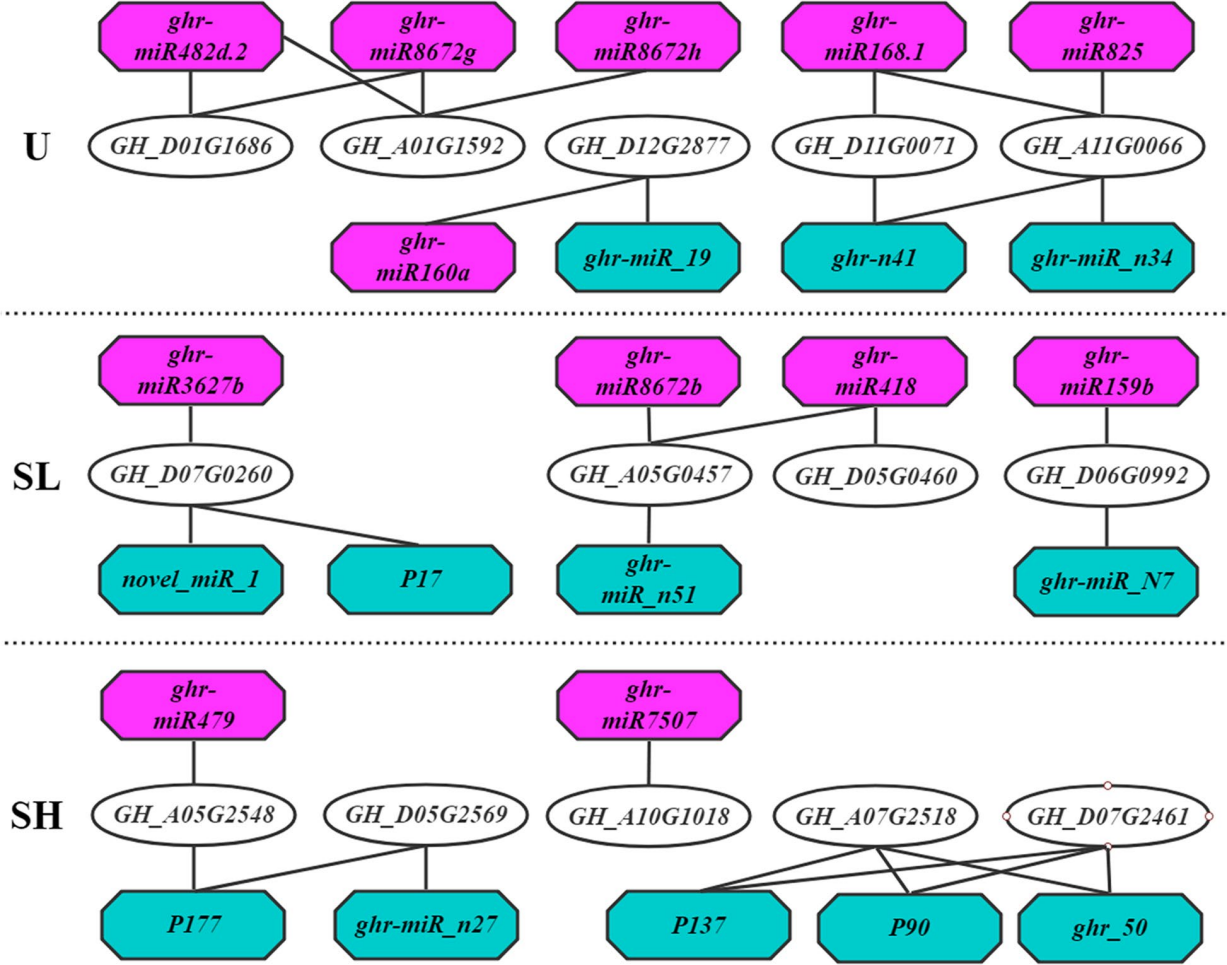
Interaction network of miRNAs and their target *GhOLEOs.* The miRNAs with pink color were the members of verified miRNA families and the miRNAs with turquoise color were newly discovered in the listed articles

To dissect the regulation of *GhOLEO* expression, the candidate miRNAs targeting the *GhOLEO*s were predicted using the psRNATarget server with the miRNAs in PMRD, miRbase, and psRNATarget databases and published miRNAs in *G. hirsutum*. With expectations lower than 4.5, 24 miRNAs targeting 14 *GhOLEOs* were identified (Fig. 4), and their details are shown in Table S5 [45]. MiRNAs and their targeted *GhOLEOs* were not one-to-one correspondence, and many miRNA targeted to a common *GhOLEO* or one miRNA had several targets. For example, *GH_D01G1686* was co-targeted by *ghr-miR482d.2* and *ghr-miR8672g*, and ghr-miR418 could suppress *GH_A05G0457* and *GH_D05G0460*. Among the 24 miRNAs, 12 (*ghr-miR159b*, *ghr-miR160a*, *ghr-miR168.1*, *ghr-miR3627b*, *ghr-miR418*, *ghr-miR479*, *ghr-miR482d.2*, *ghr-miR7507*, *ghr-miR825*, *ghr-miR8672b*, *ghr-miR8672g*, and *ghr-miR8672h*) belonged to the verified miRNA families, while others were newly discovered in the listed references (Table S6 [45–52]). In addition, five, four, and five of the *GhOLEOs* targeted by miRNA were the members of U, SL, and SH lineages, respectively.

### Expression profile analysis of *Oleosin* genes

**Fig. 5:**
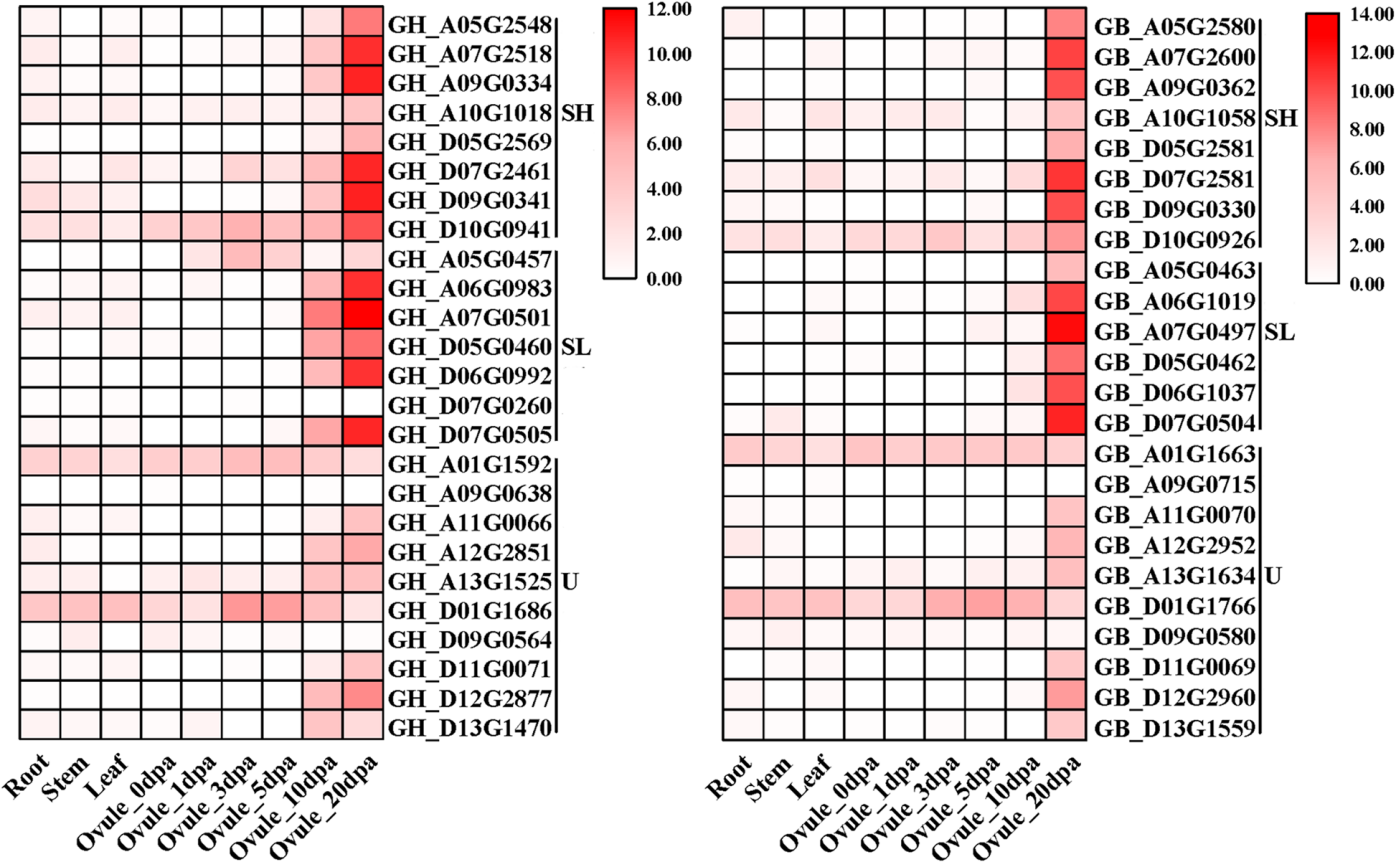
Transcriptome expression of *G. hirsutum* and *G. barbadense Oleosin* genes in root, stem, leaf, 0-20 dpa ovules

**Fig. 6:**
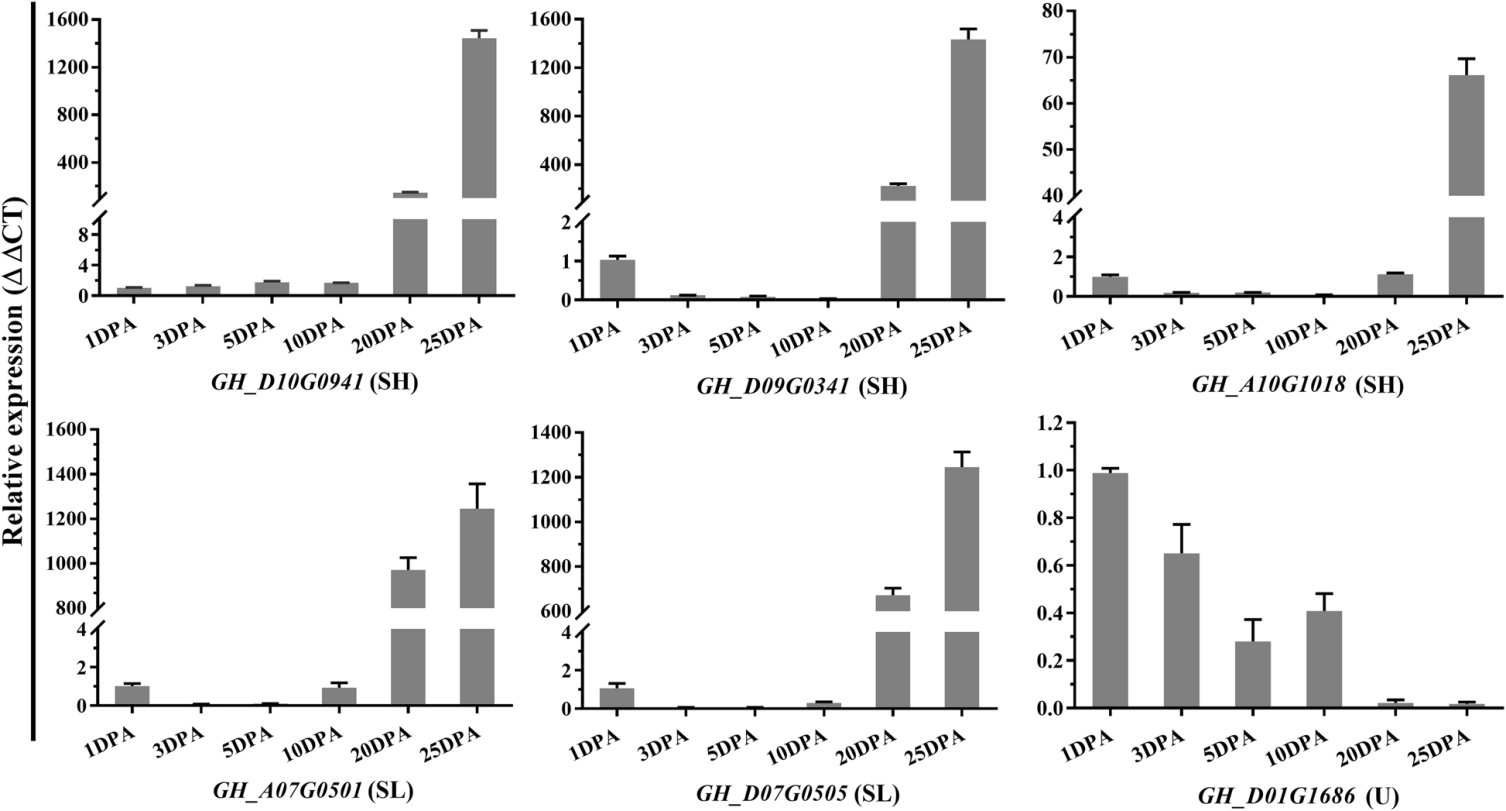
The expression levels of four *GhOLEOs* in 1 dpa, 3 dpa, 5 dpa, 10 dpa, 20 dpa, and 25 dpa ovules of *G. hirsutum* were performed with qRT-PCR. Each column of the bar chart shows the average value of three repeats, and each error bar represents one standard error

Based on the gene expression database of TM-1 and H7124 [53], the expression profiles of *OLEOs* in root, stem, leaf, and ovules (0, 1, 3, 5, 10, 20 dpa) were normalized to log2^FPKM^ and performed with heatmap (Fig. 5; Table S7). As shown, the gene expression pattern was similar between the homologous *GhOLEOs* and *GbOLEOs*. Moreover, most genes were preferentially expressed in ovules, especially in ovules of 10 and 20 dpa, indicating that these *OLEO* genes might be related to oil composition in cotton. In addition, the expression of the SL and SH *OLEOs* was higher than U *OLEOs* in ovules. In order to confirm the expression pattern of *OLEOs* in ovules, six *GhOLEO* genes with relatively high FPKM expression were further analyzed using qRT-PCR and displayed in Fig. 6. The expression pattern of these genes in qRT-PCR was very consistent with those in the heatmap. The results showed that three SH OLEOs and two SL OLEOs were highly expressed in 20 dpa and 25 dpa ovules, while *GH_D01G1686*(U) was highly expressed in 1-10 dpa ovules.

### Subcellular localization and transmembrane helices analysis of Oleosins

**Fig. 7:**
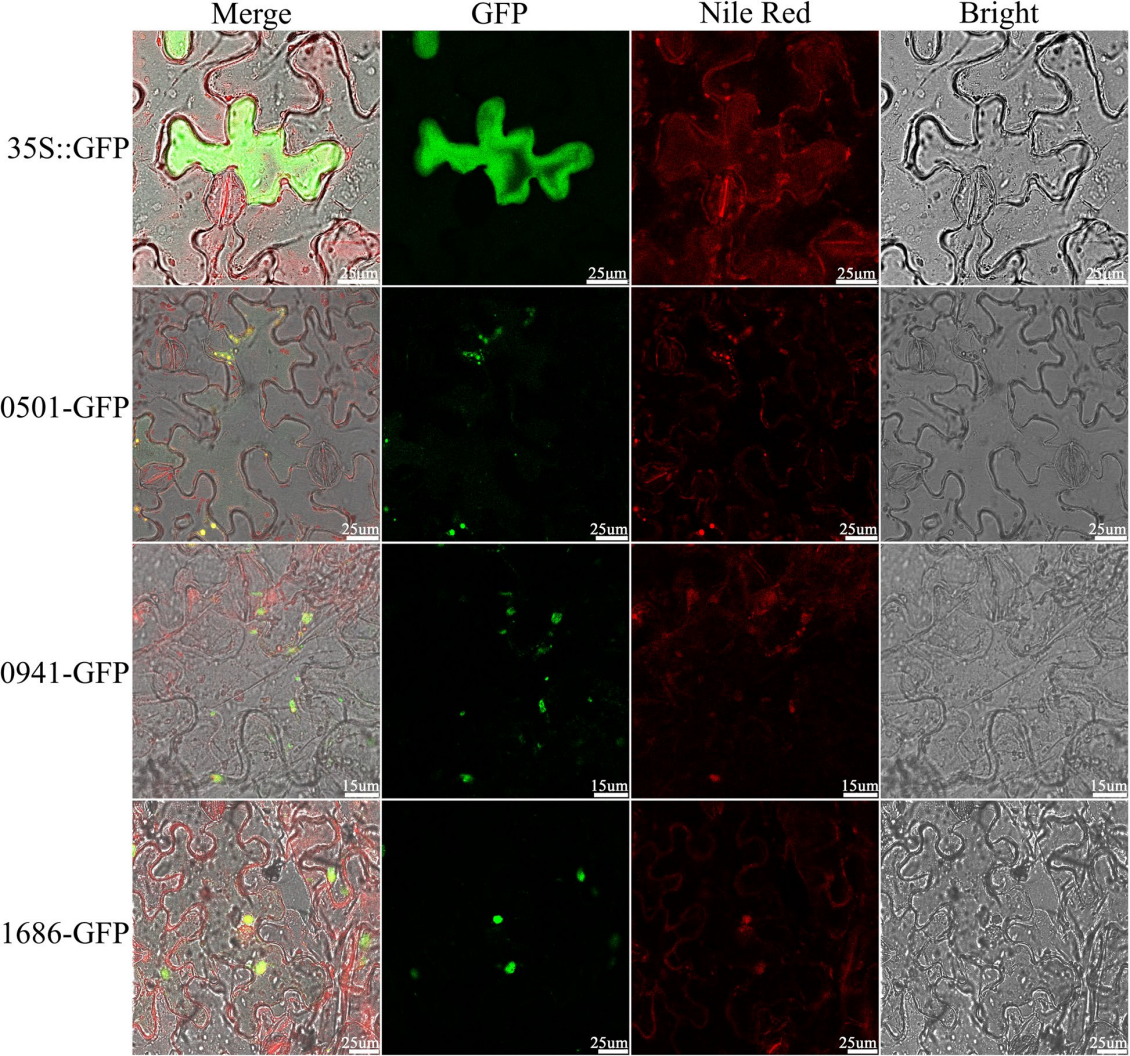
The subcellular localization of GH_A07G0501:GFP, GH_D10G0941:GFP, and GH_D01G1686:GFP in *N. benthamiana*. The empty vector of 35S::GFP was employed as control. 0501-GFP, 0941-GFP, and 1686-GFP indicated GH_A07G0501:GFP, GH_D10G0941:GFP, and GH_D01G1686:GFP, respectively. Nile red was the neutral lipid dye and emitted red light when excited by a laser

With prediction online, all the OLEO proteins in four cotton species were preliminary positioned in plasmamembrane (Table S1). To further determine the subcellular localization of *GhOLEO*s, one gene with relatively high expression at all stages was screened from SL, SH, and U. These three genes cloned from cDNA of ovules were used for constructing the GFP fusion protein. The recombinant vectors (*GH_D01G1686:GFP*, *GH_A07G0501:GFP*, and *GH_D10G0941:GFP*) were transformed into tobacco leaves, respectively, mediated by *A. tumefaciens* (EHA105). The leaves were incubated in Nile red and then were checked by monitoring the GFP signal. The lipid drops could be stained red with Nile red, and GFP proteins emit green light under excitation conditions, as shown in Fig. 7. And, the results showed that the yellow fluorescent signals, which was the composite light of green (GFP) and red (Nile red), were present in the membrane of oil bodies in the merged fields of the three fusion proteins (Fig. 7), indicating that the SL, SH, and U oleosin protein might be located on the oil body membrane (plasmamembrane).

**Fig. 8:**
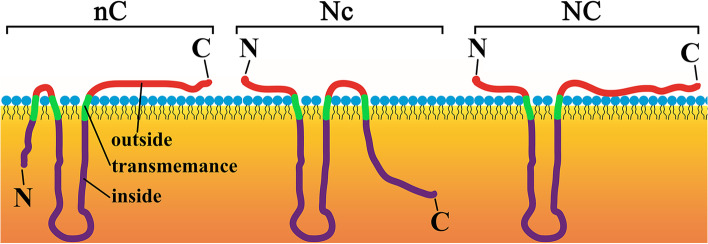
The transmembrane models of transmembrane helices in GhOLEO proteins. nC, N-terminus inside and C-terminus outside; Nc, N-terminus outside and C-terminus inside; NC, both N-terminus and C-terminus outside

For further understanding of the transmembrane helices characters, the GhOLEO protein sequences were submitted and analyzed in TMHMM Server v. 2.0 online. All the GhOLEO proteins could be categorized into three models, including nC (N-terminus inside and C-terminus outside), Nc (N-terminus outside and C-terminus inside), and NC (both N-terminus and C-terminus outside), based on the transmembrane characters (Fig. 8, Fig. S3, Fig. S4, and Fig. S5). All the nC and Nc proteins had three transmembrane helices, and one terminus (N- or C-terminus) was sticking out into the cytoplasm, while NC proteins had two transmembrane helices and both N- and C-terminus were in the cytoplasm. In SH GhOLEO proteins, only one was nC, and the others were Nc (Fig. S3), while all the SL GhOLEOs belonged to NC (Fig. S4). Furthermore, all three models appeared in the U GhOLEOs (Fig. S5).

### Ectopic overexpression of *GhOLEOs* increased seed oil content in Arabidopsis

**Fig. 9:**
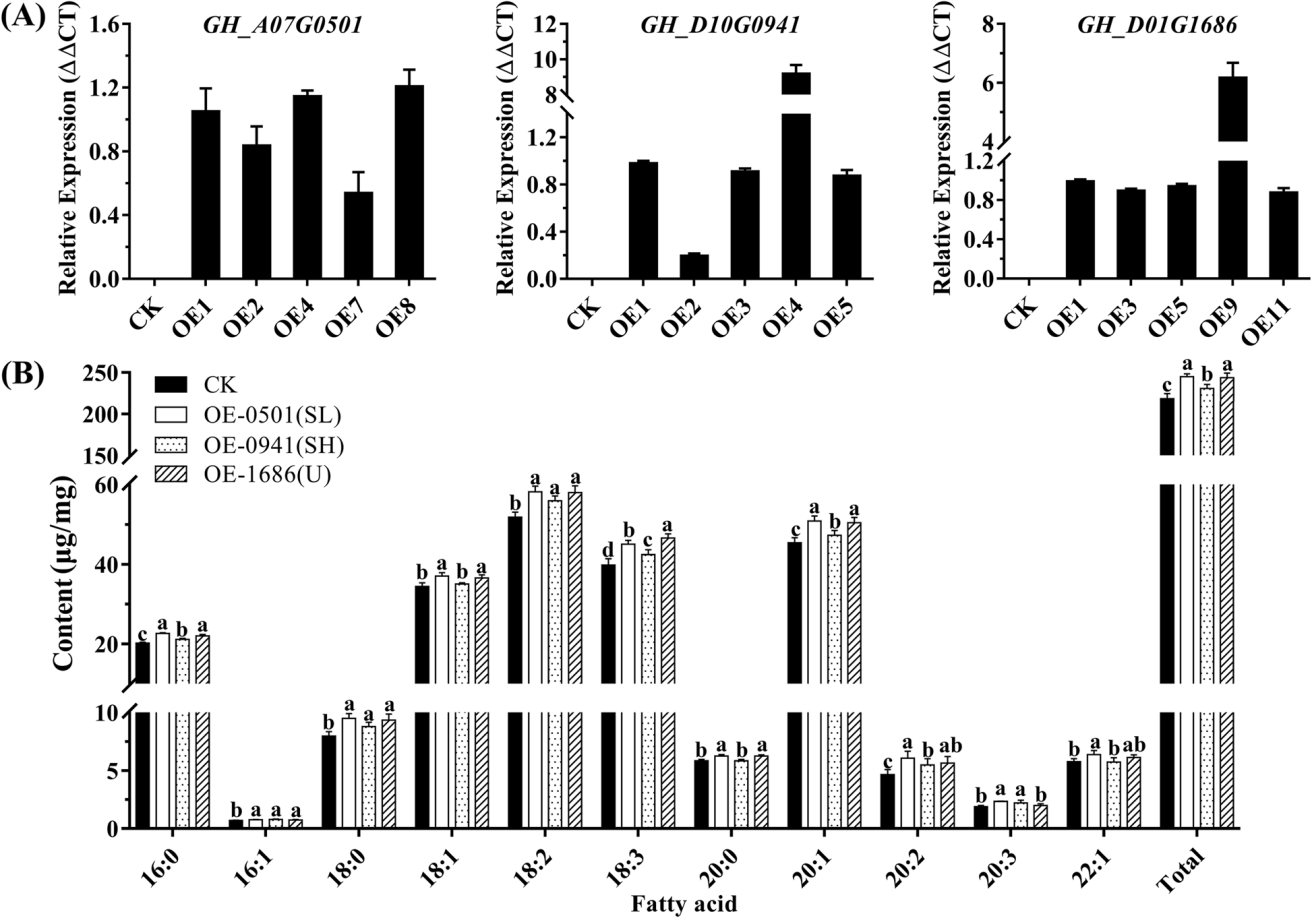
The expression levels of *GhOLEOs* and seed fatty acid contents in *GhOLEOs* transgenic *Arabidopsis* lines. **A** Gene expression levels in *Arabidopsis thaliana* transgenic lines with empty vectors (CK) and recombinant plasmids (OE). **B** The fatty acid content in CK and OE seeds detected using GC-MS methods. OE-0501, OE-0941 and OE-1686 represent the *GH_A07G0501*, *GH_D10G0941*, and *GH_D01G1686* transgenic lines. Each column of the bar chart shows the average value of three repeats, and each error bar represents one standard error

Three *GhOLEOs*, *GH_A07G0501* (SL), *GH_D10G0941* (SH), *and GH_D01G1686* (U), were cloned into vector pCAMbia2300 with 35S promoter, and the reconstructed and empty vectors were transformed into Col-0 plants. The overexpression lines (OE) and empty vector lines (CT) were selected and bred to T3 generation. Then, the expression levels of *GhOLEOs* in CT and T3 lines were analyzed by qRT-PCR (Fig. 9A). Based on their expression levels, the OE1, OE4, and OE8 of *GH_A07G0501*, the OE1, OE3, and OE5 of *GH_D10G0941*, and OE1, OE3, and OE5 of *GH_D01G1686* were employed to detect the average contents of seed fatty acid using GC-MS, and the CT lines were used as control. As shown, all the fatty acid components and their total content in seeds of *GH_A07G0501* and *GH_D01G1686* transgenic lines were significantly higher than CK lines except that 22:1 fatty acid in *GH_D01G1686* OE lines was not significantly increased (Fig. 9B). In *GH_D10G0941* transgenic lines, partial fatty acid components and the total fatty acid content were significantly increased. Moreover, in the incremental contents, the unsaturated fatty acids were 5.04 ~ 5.97 times more than saturated, and the polyunsaturated were nearly twice monounsaturated fatty acids (Table 1). These results suggested that SL, SH, and U *Oleosins* could promote the accumulation of fatty acids in seeds, and their contribution to unsaturated fatty acids was higher than saturated fatty acids.

**Table 1:** The fatty acid contents (ug/mg) in *GhOLEOs* transgenic Arabidopsis seeds

Fatty acid	CK	OE-0501(SL)		OE-0941(SH)		OE-1686(U)	
	Mean	Mean	Incremental	Mean	Incremental	Mean	Incremental
16:0	20.20	22.64	2.44	21.14	0.95	22.04	1.84
16:1	0.70	0.76	0.06	0.77	0.08	0.74	0.05
18:0	7.97	9.50	1.53	8.79	0.82	9.34	1.37
18:1	34.41	37.00	2.59	35.01	0.60	36.55	2.13
18:2	51.80	58.16	6.37	55.92	4.12	58.00	6.20
18:3	39.84	45.01	5.17	42.44	2.60	46.60	6.76
20:0	5.83	6.24	0.41	5.84	0.01	6.24	0.41
20:1	45.38	50.81	5.44	47.23	1.86	50.44	5.06
20:2	4.64	6.05	1.41	5.48	0.84	5.65	1.01
20:3	1.89	2.33	0.45	2.20	0.32	1.98	0.09
22:1	5.77	6.37	0.60	5.72	−0.04	6.13	0.36
Saturated	34.00	38.38	4.38	35.77	1.78	37.62	3.63
Monounsaturated	86.25	94.94	8.69	88.74	2.49	93.85	7.60
Polyunsaturated	98.17	111.56	13.39	106.04	7.88	112.24	14.07
unsaturated	184.41	206.50	22.08	194.78	10.37	206.09	21.67
Total	218.41	244.88	26.47	230.55	12.14	243.71	25.30

Incremental indicates the increased contents of fatty acids compared to the control group

### *GhOLEOs* decrease the germination rates of seeds

**Fig. 10:**
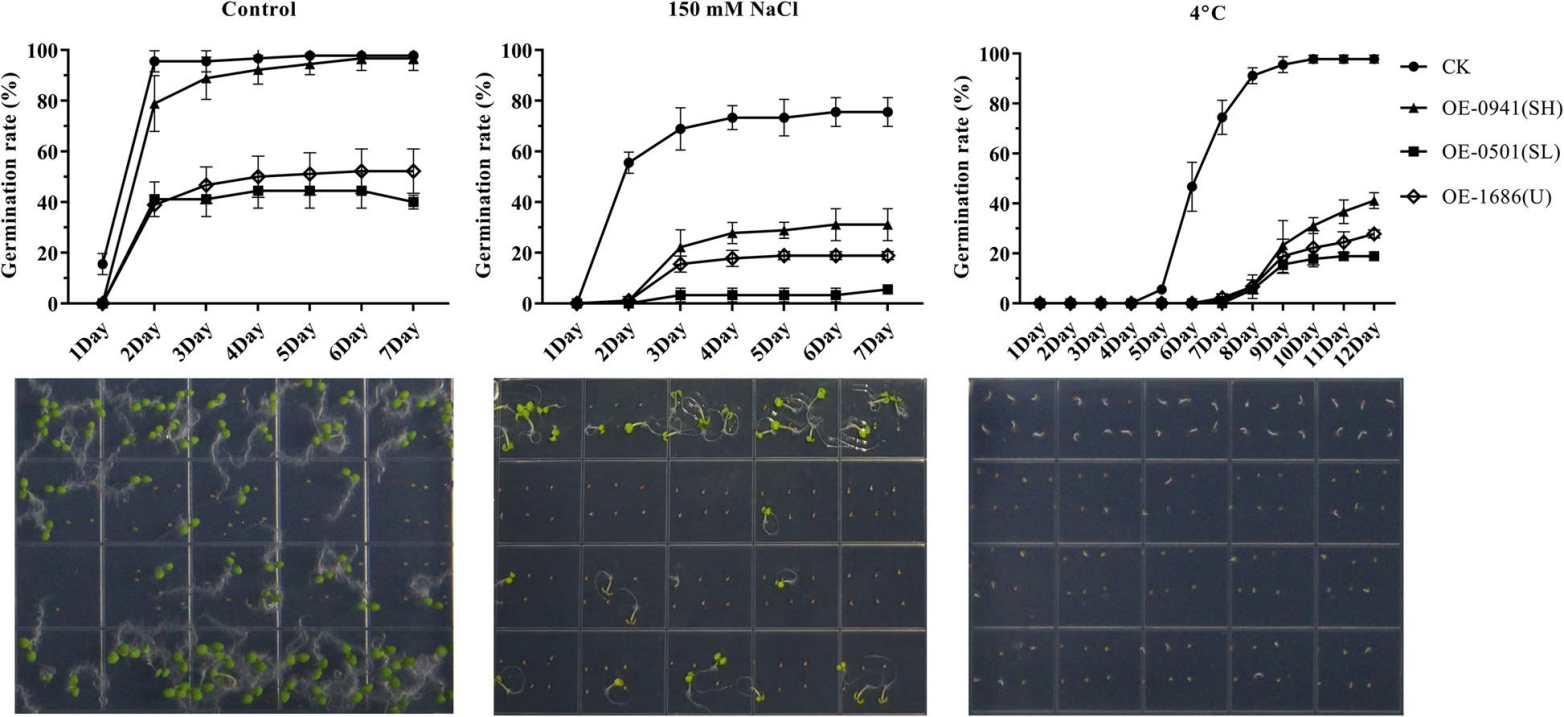
The germination rates of *GhOLEOs* transgenic Arabidopsis lines under the normal, 150 mM NaCl, and 4 °C chilling conditions. The line charts showed the statistical results of germination rate in three independent biological replicates, and the corresponding photos on the last statistical date (Control and 150 mM NaCl: seventh Day; 4 °C: twelfth Day) were performed below. Each point shows the average value of three repeats, and each error bar represents one standard error. OE-0501, OE-0941, and OE-1686 represent the *GH_A07G0501* (SL), *GH_D10G0941* (SH), and *GH_D01G1686* (U) transgenic lines of *Arabidopsis thaliana*

As is known, fatty acids play an important role in the resistance of plants to stress, such as chilling, salt, and drought. Moreover, the lipids could provide the seeds with energy to survive adverse conditions. Previous research suggested that oleosin, maintaining seed oil content and oil body size, was essential in the resistance of seeds to freezing [54, 55]. So, we asked whether the oleosin could increase the resistance of seeds to salt and chilling. For this, the germination rates (GR) of the OE lines selected above and the CT lines were determined under control, 150 mM NaCl, and 4 °C conditions, respectively. The results showed that the GRs of CT and OE lines performed better under control conditions than at 150 mM NaCl and 4 °C. The GRs of CT, OE-GH_D10G0941 (SH), OE-GH_D01G1686 (U), and OE-GH_A07G0501 (SL) were successively from high to low in all conditions (Fig. 10). These results indicated that the overexpression of *GhOLEOs* decreases the germination rates of seeds and the resistance to salt and chilling.

## Discussion

The *OLEO* gene family has been whole-genome screened and characterized in several plants, including *Coffea canephora* [56], *Brassica napus* L [17], and *Carthamus tinctorius* L [57]. Their functions were also widely studied in many plant species with cloning and overexpression [31, 33]. It is generally accepted that oleosins play an essential role in OB regulation and lipid accumulation [17, 27–31]. However, it is paid little attention to the oleosins in cotton, which is also a critical oil crop. Nowadays, the genomes of *G. hirsutum*, *G. barbadense*, *G. arboreum*, and *G. raimondii* have been better sequenced further to understand cotton genomics and genetics [53, 58, 59]. With this, the members of the *OLEO* gene family and their evolutionary relationships might be further explored and determined in cotton.

In this study, a total of 25, 24, 12, and 13 *OLEOs* were identified in *G. hirsutum*, *G. barbadense*, *G. arboreum*, and *G. raimondii,* respectively (Table S1)*.* In the previous studies, *OLEOs* had been clustered into six lineages (M, T, P, U, SL, and SH) with the alignment of about 1000 oleosin sequences in 22 species [14]. Moreover, T lineage in the terrestrial lineages (T, U, SL, and SH) existed only in Tapeta. The cotton *OLEOs* were divided into U, SL, and SH lineages, according to the *OLEO* phylogenetic tree combing *A. thaliana, B. napus,* and four cotton species (Fig. 1). For the number of *OLEOs* in each lineage, each tetraploid cotton had nearly twice as many as each diploid cotton, and the *OLEOs* of tetraploid corresponded to *OLEOs* of diploid one by one and were clustered together at the end branches of the evolutionary tree. These results also indicated that the *OLEO* gene families of the A- and D-genome in diploids combined and formed the AD gene family in neoallopolyploids, which occured during the polyploidization of two diploid cotton species to form the allopolyploid cotton approximately 1.7-1.9 million years ago [53].

Studying the gene structure is necessary to identifying gene evolution. In the present study, motif 1 containing proline junction (PX_5_SPX_3_P) was common to all *OLEO* genes except *GB_A09G0715*, which also had a proline junction. The proline junction (PX_5_SPX_3_P) was the loop of the highly conserved hairpin structure, which penetrates into the core of the oil body and plays an essential role in targeting and stabilizing the oil body [18]. In contrast, some conserved motifs are specific to subfamilies in oleosin proteins of cotton. The specific conserved motifs and gene structure possibly resulted in functional differences of *OLEOs* in different evolutionary tree lineages. For instance, motifs 7, 12, 13, 16, 20, 22, and 23 were specific to SH *OLEOs*, which might lead to conserved and specific functions in these genes. Gene duplication is the main driver of evolution, increasing the gene number and leading to functional differentiation for adapting to environmental changes [60]. In this study, all *OLEOs* in tetraploid kinds of cotton and most *OLEOs* in diploids were replicated through WGD or segmental duplications, while several *OLEOs* of diploid kinds of cotton were dispersed duplicated (Table S3).

MiRNAs, 19-24 bp length non-coding RNAs widespread in plants, animals, and viruses, play significant roles in post-transcriptional regulation. Likewise, they also significantly contributed to plant developments, including fatty acid biosynthesis [61]. For dissecting the regulation of *GhOLEO* expression, 24 candidate miRNAs targeting the *GhOLEO*s were predicted using the psRNATarget server (Fig. 4), and 12 of them belonged to 10 verified miRNA families, including *ghr-miR159*, *ghr-miR160*, *ghr-miR168*, *ghr-miR3627*, *ghr-miR418*, *ghr-miR479*, *ghr-miR482*, *ghr-miR7507*, *ghr-miR825*, and *ghr-miR8672*. Studies have shown that *miR159* plays important roles in fatty acid biosynthesis during seed development in sea buckthorn, soybean, rice, *Brassica napus*, Arabidopsis [62–66]. Also, miR160 significantly affected seed development in *A. thaliana* [67]. The identification of ghr-miRNA genes and their targets broadens our understanding of the regulatory mechanism of *GhOLEOs* involved in cotton fatty acid biosynthesis.

Oleosins were proved to be the main structural proteins in the oil bodies, which have a core of neutral lipids surrounded by the phospholipid monolayer [4, 6]. In cotton, the oleosins were predicted to bind to plasmamembrane (Table S1). Moreover, the subcellular localization of GH_D01G1686:GFP, GH_A07G0501:GFP, GH_D10G0941:GFP, and GFP proteins showed the SL, SH, and U oleosins were anchored to the oil body membrane (Fig. 7), which was consistent with previous results. To further understand the mode in which the oleosins bind to the membrane, the transmembrane helices of all GhOLEO proteins were analyzed using web-server TMHMM 2.0. And, all the transmembrane helices characters were observed and summarized into three transmembrane models, consist of nC, Nc, and NC (Fig. 8, Fig. S3, Fig. S4, and Fig. S5). It was found that the SH oleosins were nC or Nc models, in which three transmembrane helices existed, and only one terminus was outside to the cytoplasm (Fig. 8 and Fig. S3). In addition, all the SL oleosins were NC models, whose both N- and C-terminus were outside, while the U oleosins contained all the three models (Fig. S4 and Fig. S5). However, only the NC model, both N-terminus and C-terminus outside, was reported in previous researches [14, 15, 20]. These three speculated models, especially the newly proposed nC and Nc models, should be verified experimentally and might provide a reference for the further study of gene function.

Previous studies have shown that oleosins contribute significantly to the formation of oil bodies and the accumulation of lipids [7, 17, 28, 31]). In this research, the contribution of *SL-*, *SH-*, and *U-OLEOs* to lipid accumulation in seeds were investigated with overexpressing *GhOLEOs* in Arabidopsis*.* As shown in Fig. 9, all the fatty acid components were increased in Arabidopsis seeds with overexpression of SL, SH, or U *GhOLEOs.* Although previous results showed that the fatty acid or oleosins could increase the stress resistance of seeds [68], the overexpression of *GhOLEOs* in Arabidopsis leaded to a low germination rate and resistance of seeds under normal, salt, or chilling condition (Fig. 10). The oleosins, no matter SH, SL, and U, play an essential role in maintaining the stability of the oil body [21, 69]. Their degradation is involved in lipids release from oil bodies during germination [70, 71]. The increase of oleosins might enhance the binding force of the oil body on lipids, and the lipid mobilization became harder. The overexpression of oleosin decreased the size of the oil body and increase the content of lipids in seeds [31, 33], while the loss of oleosin led to bigger oil bodies and lower lipid content in seeds [7, 32]. Furthermore, the oil body size was partly controlled by the ratio of lipid to oleosin proteins [72]. So, the oleosin was indispensable but not too much. Thus, the germination and stress resistance of seeds with higher oleosin levels became lower in this study. In addition, the co-expression of oleosin with lipid synthesis genes, such as *DGAT*, *WRI1,* and *FAD*, could further improve the oil content in seeds [17, 73]. And, this method might recover the ratio of lipid to oleosin and the oil body size compared with the seeds of transgenic lines that only expressed the single gene *OLEO*. These results might provide a foundation or reference for the development of cultivars with high oil and improvement of the lipid content in cotton seeds.

## Conclusions

In this research, 25 *GhOLEOs*, 24 *GbOLEOs*, 12 *GaOLEOs,* and 13 *GrOLEOs* were identified and clustered into three lineages according to the phylogenetic tree. Then, their chromosomal location, gene structure, conserved motifs, conserved domains, and collinearity and duplication were analyzed for understanding the gene family expansion and gene evolution. Synteny analysis revealed that most of the oleosin genes were conserved, and their expansion might be mainly driven by WGD or segmental duplications. With bioinformatics tools, 24 candidate miRNAs targeting *GhOLEOs* were obtained, and the miRNA-*GhOLEO* regulatory network was constructed. The transmembrane helices in GhOLEO proteins were predicted, and three transmembrane models were summarized. In addition, the expressions of *GhOLEO* in different tissues and developmental stage ovules were performed and confirmed using qRT-PCR, implying that *GhOLEOs* might be involved in oil accumulation. Furthermore, their functions involved in oil accumulation and germination of seeds were identified with overexpressed in Arabidopsis and calculating germination rate of transgenic seeds under normal, salt, and chilling conditions. Take together, these findings provide insight into the potential functional roles of the *GhOLEO* genes and will help to improve the seed oil content of *G. hirsutum*.

## Methods

### Plant materials and sequence retrieval

The 1-25 dpa (days post-anthesis) ovules of ‘Han682’ cotton (*G. hirsutum*) plants, originally cultivated by Handan Academy of Agricultural Sciences, were harvested from the experimental field of Shandong Agricultural University [74]. The samples were immediately frozen in liquid nitrogen and stored at − 80 °C. These plant materials sampled for experimental research required no permissions. Experimental research on plants in this study, including the collection of plant material, comply with institutional, national, or international guidelines and the Convention on the Trade in Endangered Species of Wild Fauna and Flora.

The genome files and annotation gff3 files of *G. hirsutum* (ZJU, TM-1), *G. barbadense* (ZJU, H7124), *G. arboreum* (CRI, Shiyaxi1), and *G. raimondii* (JGI) were downloaded from the Cottongen database (https://www.cottongen.org). The OLEO protein sequences of *Arabidopsis thaliana* [75] and *Brassica napus* [17] were obtained from the EnsemblPlants database (http://plants.ensembl.org/Brassica_napus/Info/Index) and TAIR database (http://www.arabidopsis.org/index.jsp), respectively.

### Genome-wide identification and properties analysis of *Oleosin* genes

Firstly, all the 17 *A. thaliana* OLEO proteins and 48 *B. napus* OLEO proteins were used as query sequences to scan the whole genome protein sequences of four cotton species with BLASP search (e-value <1e-5). Secondly, the Hidden Markov Model (HMM) profile of the Oleosin domain (PF01277), acquired from the Pfam database (http://pfam.xfam.org/), was used in HMM search to identify *OLEO* genes. The conversed protein sequence of oleosins with e-value <1e-15 were aligned using ClustalW and used to construct the new HMM profile for each cotton species. The species-specific HMM profiles were then employed to detect possible oleosins in each cotton species. Finally, all the non-redundant protein sequences, from the results of BLASTP and HMM search, were further identified using the NCBI Conserved Domain Database (CDD, https://www.ncbi.nlm.nih.gov/cdd) with the automatic model and default parameters (threshold = 0.01, maximum hits =500).

The information about all oleosins, including physical location, strand, length, coding sequence (CDS) length, exon number, the number of amino acids (NNA), molecular weight (Mw), charge, isoelectric point (pI), and grand average of hydropathy (GRAVY) were fetched with feature analysis in CottonFGD database. The chromosome localization of *OLEO* genes was drawn using TBtools [76] with the genome annotation gff3 files.

### Duplication and synteny analysis of *Oleosin* genes

Firstly, the whole genome protein sequences of the four cotton species were pairwise compared using BLAST. Then, the synteny examination of paralogous genes in four cotton species was calculated with MCScanX (http://chibba.pgml.uga.edu/duplication/) in TBtools. Afterward, synteny visualization was conducted using TBtools.

### Phylogenetic analysis of *Oleosin* genes

The phylogenetic tree was constructed with Mega X [77] as follows: the protein sequences were aligned using ClustalW with default parameters; after that, the Maximum Likelihood tree was built with the Poisson model, and 1000 replicates bootstrap. The tree was colored in web-server ITOL (https://itol.embl.de/).

### Gene structure and conserved motif analysis

Gene Structure Display Server 2.0 (GSDS, http://gsds.cbi.pku.edu.cn/) was employed to analyze the exon-intron structures of the *OLEO* genes. And, the conserved motifs of the oleosins were detected using MEME v5.1.0 (http://meme-suite.org/tools/meme) with parameters as follow: zoop (zero or one occurrence per sequence) was selected in site distribution, the width of motifs was 6 to 50, and the maximum number of motifs was set as 24. The visualization of all the characteristic results was constructed and merged in TBtools.

### Prediction of miRNA targeting *GhOLEO* genes

The miRNAs targeting *GhOLEO* genes were predicted by querying their full coding sequences against the miRNA databases in web-server PMRD (plant microRNA database, https://bioinformatics.cau.edu.cn/PMRD/), miRbase (http://www.mirbase.org/), and psRNATarget (http://plantgrn.noble.org/psRNATarget/analysis?function=2), and the non-redundant miRNAs identified in published papers (Table S6 [45–52];). The default parameters used were modified with the maximum expectation to 4.5. *GhOLEOs*-miRNAs interaction networks were illustrated using the Cytoscape 3.7.0 software [78].

### Expression analysis of *Oleosin* genes

Based on the gene expression database of TM-1 and H7124 [53], the expression profiles of *OLEOs* in root, stem, leaf, and ovules (0, 1, 3, 5, 10, 20 dpa) were obtained and analyzed. The expression of *OLEOs* was displayed in the heatmap after normalized with log_2_(FPKM+ 1). Furthermore, the expressions of *GhOLEO* genes were checked with qRT-PCR of 1, 3, 5, 10, 20, and 25 dpa ‘Han682’ ovules. The total RNA was extracted with an OminiPlant RNA Kit (DNase I) (CWBIO). A 20-μL reaction volume containing 1 μg of total RNA was used to synthesize template cDNA with a HiFiScript cDNA Synthesis Kit (CWBIO). In the qRT-PCR, *UBQ7* was used as an internal reference, and the Applied Biosystems 7500 Real-Time PCR System was employed. Each reaction was performed at least three times, and relative expressions were analyzed with the ΔΔCt method. All the qRT-PCR primers were shown in Table S8.

### Subcellular localization and overexpression of *GhOLEOs*

The subcellular localization of all *OLEOs* in four cotton species was predicted in the web-server CELLO v2.5 (http://cello.life.nctu.edu.tw/). For experimental verification of these predicted results, the non-terminator coding sequences of three *GhOLEOs* in SL, SH, and U lineages were cloned from cDNA of Han682, which was cultivated by Handan Academy of Agricultural Sciences, and fused to a green fluorescent protein (GFP) into vector pBI121 for subcellular localization. The specific primers were listed in Table S8. The recombinant plasmid and the control pBI121-35S::GFP *Agrobacterium tumefaciens* (EHA105) cells were injected into *Nicotiana benthamiana* leaves (4 weeks old). After being cultured in dark overnight and 16 h light/8 h dark photoperiod 2 d, the leaves selected were incubated in the Nile red for 15 min and washed in water for 5 min before scanning. Then, the leaves were observed and photographed with an Agilent TCSsp5IIconfocal laser scanning microscope. GFP was excited with the 488 nm line of an argon laser and the Nile red with a 633 nm neon laser. The detection of GFP and Nile Red emission was performed in a sequential line-scanning mode with 495-510 nm and 637-650 nm, respectively. The subcellular localization of *GhOLEOs* was observed by combing CLSM and bright-field microscopy. Prediction of transmembrane helices in proteins was completed in TMHMM Server v. 2.0 (http://www.cbs.dtu.dk/services/TMHMM/).

In addition, the full coding sequences of *GhOLEO* genes were cloned into vector pCAMbia2300 with the 35S promoter, respectively, for gene overexpression. The specific primers were listed in Table S8. The reconstructed plasmids and empty vectors above were introduced into *A. tumefaciens* (EHA105), respectively. Then, the plasmids were transformed into *A. thaliana* wild-type (Col-0, WT) plants using the floral dip method [79]. The empty vector transgenic lines were used as control (CK).

### Seed oil content determination

The washed seeds (10 mg) were placed into a glass tube containing 1.5 mL of 2.5% sulfuric acid-methanol solution, 0.3 μg of butylated hydroxytoluene, and 0.25 mL of toluene. Then, 0.1 mL of C19:0 in toluene (2.2 mg/mL) was added to the tube as a standard internal substance. After vortexed, the mixture was heated in water at 90 °C for 1 h and then cooled. The solution was left to stand overnight after adding 1.6 mL of ddH_2_O and 1 mL of hexane. Afterward, the supernatant was filtered using a 0.45-μm microporous membrane. Then 1 mL filtrate was used to detect the oil content by GC-MS. The parameters for GC-MS were as follows: an Agilent 7890A-88HP-INNOWAX column (100 m × 0.25 mm × 0.25 mm) was employed; the flow rate of Helium (carrier gas) was kept at 1 mL/min; the column temperature was held at 120 °C for 5 min firstly, and then raised to 240 °C with 3.5 °C/min and maintained for 10 min; the interface temperature of GC-MS was set to 250 °C, the ion source temperature was at 230 °C and EI ionization at 70 eV.

### Salt /chilling tolerance assessment

For germination assessment, the Arabidopsis (Col-0) seeds were sterilized and then sown on 1/2 Murashige and Skoog (MS) medium containing 0 mM NaCl (Control, CT) or 150 mM NaCl (NaCl treatment). After vernalization for 4 d at 4 °C and darkness, the plates were cultured at 22 °C and 12 h light/12 h dark. Simultaneously, some plates without NaCl were placed at 4 °C and 12 h light/12 h dark (marked as Chilling treatment). The germinated seeds (CT and NaCl treatment) were counted on 1-7th day after sowing for salt tolerance assessment. CT and Chilling treatment were counted on the 1-12th day after planting for chilling tolerance assessment.

### Statistical analysis of data

For the accuracy of the experiment, data detection and measurement were repeated more than three times in this study. Finally, the mean values and the standard deviations of repeats were presented.

## Supplementary Information

**Additional file 1: Figure S1** Chromosome distribution of *Oleosin* genes in four cotton species. **Figure S2** Collinearity analyses of *Oleosin* genes between *G. hirsutum*, *G. barbadense*, *G. arboretum,* and *G. raimondii.* **Figure S3** The prediction of transmembrane helices in SH GhOLEO proteins. nC, N-terminus inside and C-terminus outside; Nc, N-terminus outside and C-terminus inside. **Figure S4** The prediction of transmembrane helices in SL GhOLEO proteins. NC, both N-terminus and C-terminus outside. **Figure S5** The prediction of transmembrane helices in U GhOLEO proteins. nC, N-terminus inside and C-terminus outside; Nc, N-terminus outside and C-terminus inside; NC, both N-terminus and C-terminus outside.

**Additional file 2: Table S1** The sequence properties of *Oleosin* genes identified in four cotton species. **Table S2** The homologous *Oleosin* genes among four cotton species. **Table S3** Duplicated *Oleosin* gene pairs in each cotton species. **Table S4** Information of motifs in *Oleosin* genes. **Table S5** Details for predicted miRNA targeting *GhOLEOs*. **Table S6** The non-redundant published miRNAs used in this study. **Table S7** The expression profiles (FPKM) of *OLEOs* in root, stem, leaf, and 0-20 ovules [53]. **Table S8** Specific primers for qRT-PCR or vector constructions.

## Data Availability

The genome files and annotation gff3 files of *G. hirsutum* (ZJU, TM-1), *G. barbadense* (ZJU, H7124), *G. arboreum* (CRI, Shiyaxi1), and *G. raimondii* (JGI) were downloaded from the Cottongen database (https://www.cottongen.org). The OLEO protein sequences of *Arabidopsis thaliana* and *Brassica napus* were obtained from the EnsemblPlants database (http://plants.ensembl.org/Brassica_napus/Info/Index) and TAIR database (http://www.arabidopsis.org/index.jsp), respectively. Experimental research on plants in this study complies with institutional, national, or international guidelines and the Convention on the Trade in Endangered Species of Wild Fauna and Flora.

## Abbreviations

*OLEO*: *Oleosin*
TAGs: Triacylglycerols
OBs: Oil bodies
ER: Endoplasmic reticulum
M: Mesocarp lineage
T: Tapetum lineage
P: Primitive lineage
U: Universal lineage
SL: Seed low-molecular-weight lineage
SH: Seed high-molecular-weight lineage
GR: Germination rate
NAA: The number of amino acids
Mw: Molecular weight
pI: Isoelectric point
WGD: Whole-genome duplications
HMM: Hidden Markov Model
CDD: Conserved domain database
GRAVY: Grand average of hydropathy
