# Supplementary material for: Genome-wide identification and analysis of Oleosin gene family in four cotton species and its involvement in oil accumulation and germination
Source: BMC Plant Biol. 2021 Dec 4;21:569. doi: 10.1186/s12870-021-03358-y (PMC8642851; doi:10.1186/s12870-021-03358-y)
Supplement: Supplementary file 1 — Additional file 1: Figure S1 Chromosome distribution of Oleosin genes in four cotton species. Figure S2 Collinearity analyses of Oleosin genes between G. hirsutum, G. barbadense, G. arboretum, and G. raimondii. Figure S3 The prediction of transmembrane helices in SH GhOLEO proteins. nC, N-terminus inside and C-terminus outside; Nc, N-terminus outside and C-terminus inside. Figure S4 The prediction of transmembrane helices in SL GhOLEO proteins. NC, both N-terminus and C-terminus outside. Figure S5 The prediction of transmembrane helices in U GhOLEO proteins. nC, N-terminus inside and C-terminus outside; Nc, N-terminus outside and C-terminus inside; NC, both N-terminus and C-terminus outside. [file 12870_2021_3358_MOESM1_ESM.pdf]

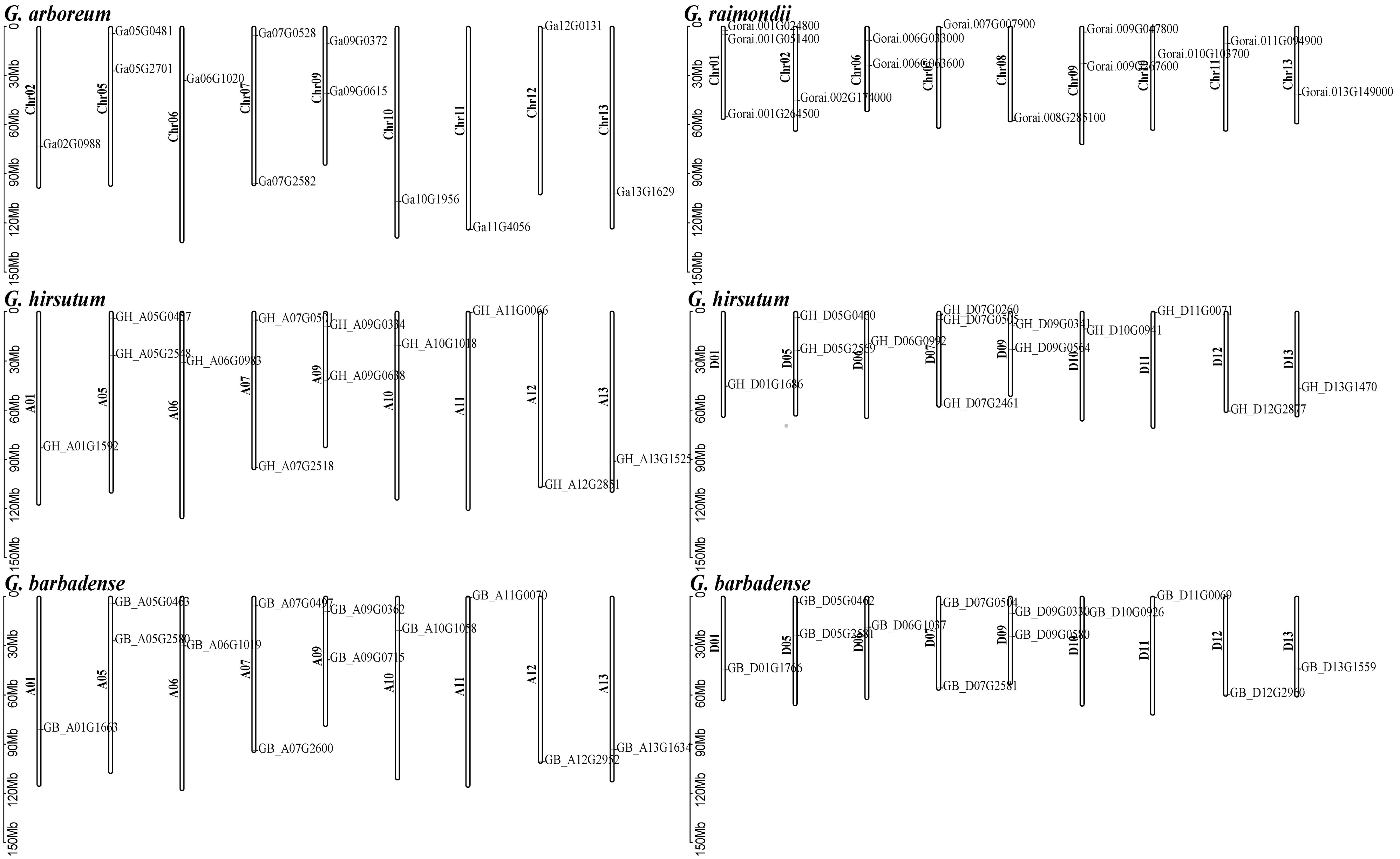

Figure S1 Chromosome distribution of *Oleosin* genes in four cotton species.

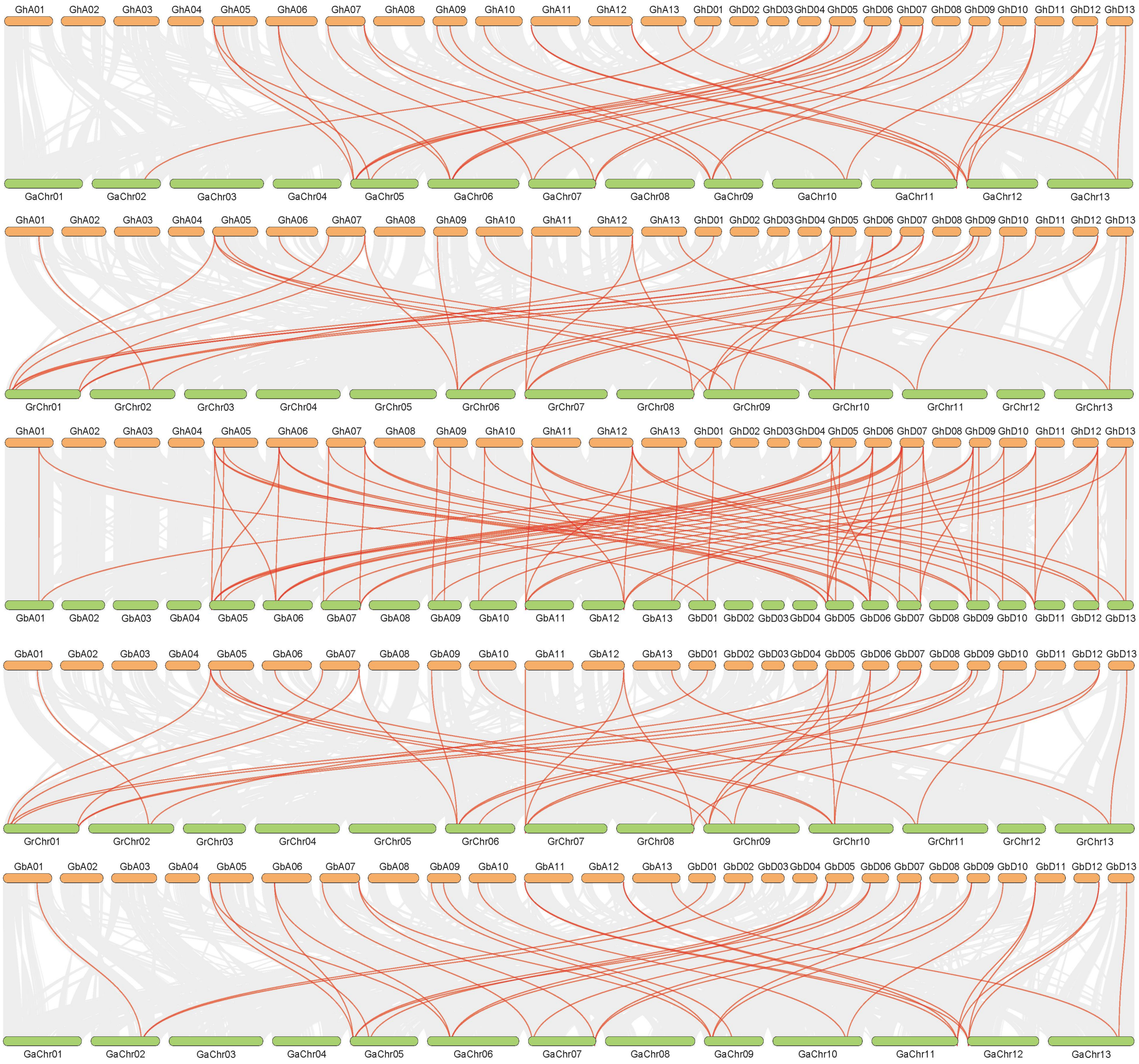

Figure S2 Collinearity analyses of *Oleosin* genes between *G. hirsutum*, *G. barbadense*, *G. arboretum*, and *G. raimondii.*

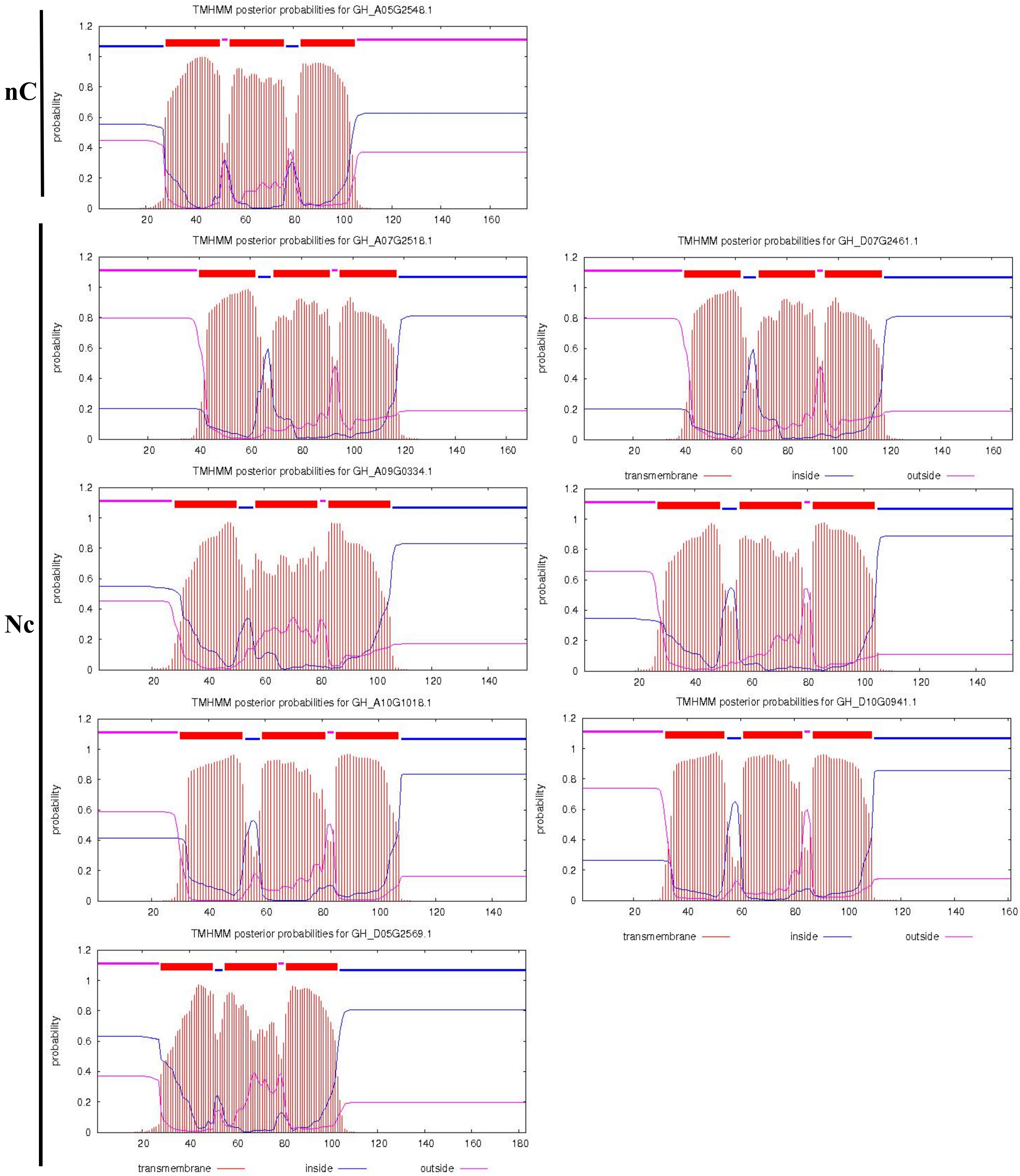

Figure S3 The prediction of transmembrane helices in SH GhOLEO proteins. nC, N-terminus inside and C-terminus outside; Nc, N-terminus outside and C-terminus inside.

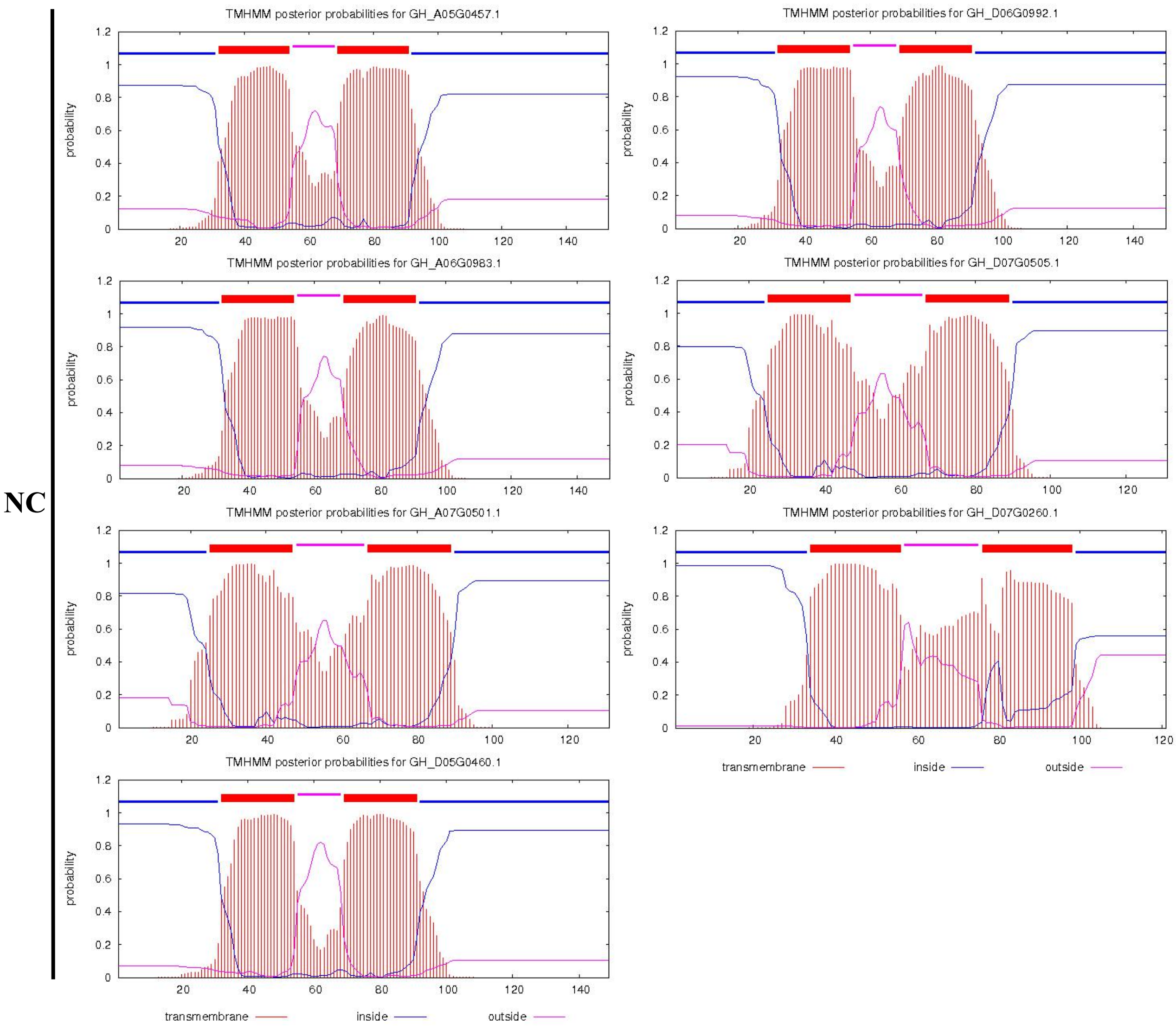

Figure S4 The prediction of transmembrane helices in SL GhOLEO proteins. NC, both N-terminus and C-terminus outside.

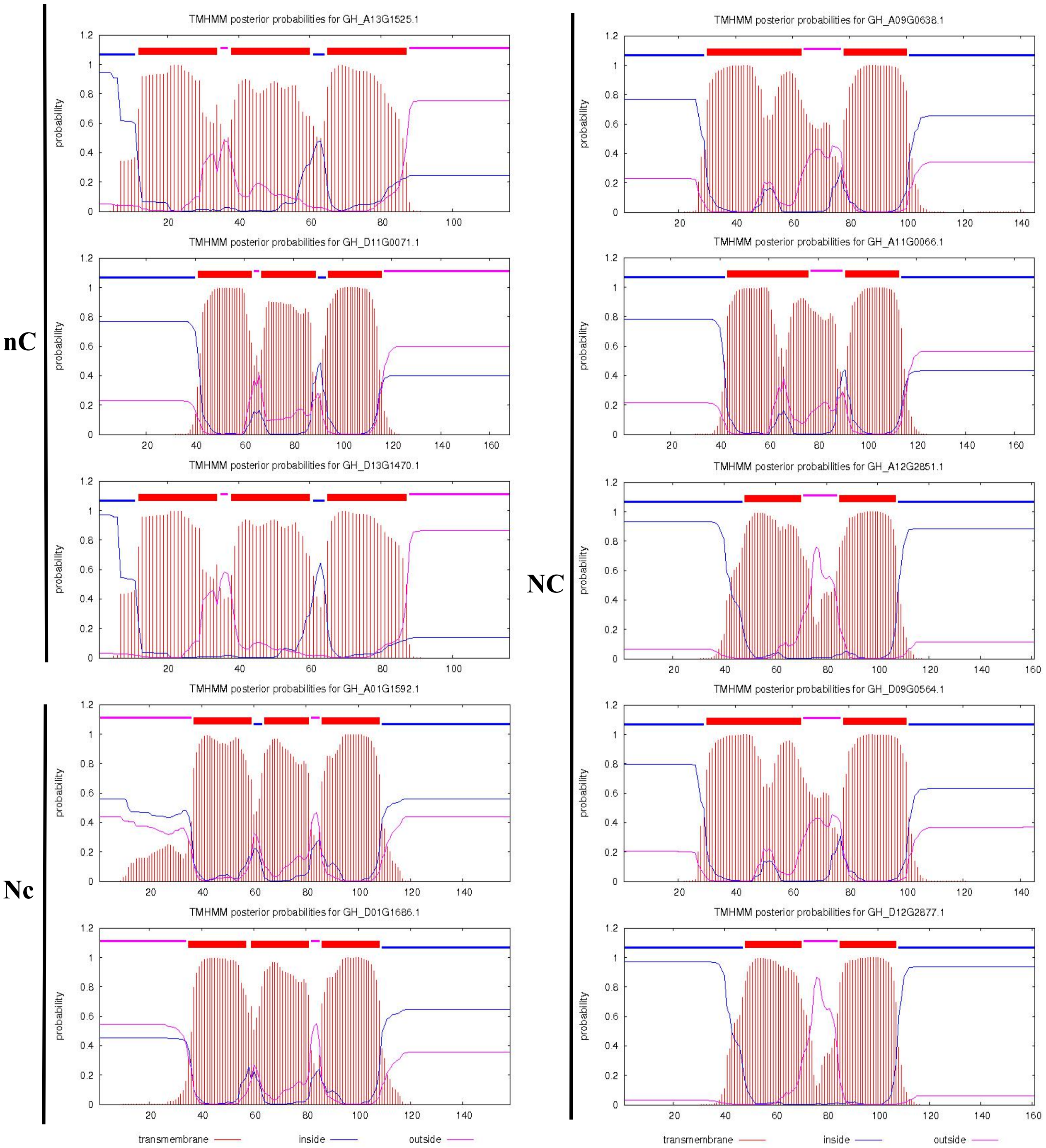

Figure S5 The prediction of transmembrane helices in U GhOLEO proteins. nC, N-terminus inside and C-terminus outside; Nc, N-terminus outside and C-terminus inside; NC, both N-terminus and C-terminus outside.
